# Gene network analysis reveals a novel 22-gene signature of carbon metabolism in hepatocellular carcinoma

**DOI:** 10.18632/oncotarget.10249

**Published:** 2016-06-23

**Authors:** Jinqiang Zhang, Melody Baddoo, Chang Han, Michael J. Strong, Jennifer Cvitanovic, Krzysztof Moroz, Srikanta Dash, Erik K. Flemington, Tong Wu

**Affiliations:** ^1^ Department of Pathology and Laboratory Medicine, Tulane University School of Medicine, New Orleans, Louisiana, USA; ^2^ Bioinformatics Core, Tulane Health Sciences Center and Tulane Cancer Center, New Orleans, Louisiana, USA; ^3^ Biospecimen Core, Louisiana Cancer Research Consortium, New Orleans, Louisiana, USA

**Keywords:** transcriptome, carbon metabolism, weighted gene coexpression network analysis (WGCNA), citrate synthase (CS), Acetyl-CoA synthetases short-chain family member 1 (ACSS1)

## Abstract

Although much progress has been made in understanding cancer cellular metabolism adaptation, the co-regulations between genes of metabolism and cancer pathways and their interactions remain poorly characterized. Here, we applied gene co-expression network analysis to 1509 metabolic gene expression data generated from 120 HCC and 180 non-tumor human liver tissues by microarray. Our analyses reveal that metabolism genes can be classified into different co-expression modules based on their associations with HCC related traits. The co-regulation mechanism of the carbon metabolism genes in normal liver tissues was interrupted during the processes of carcinogenesis. In parallel, we performed RNAseq analysis of HCC and non-tumor human liver tissues, and identified a unique 22-carbon-metabolism-gene-signature of increased expression. This gene signature was further verified in multiple microarray data sets, and its prognostic value was also proven by HCC patients' survival data from TCGA. Additionally, the tumorigenic function of two representative genes, CS and ACSS1, were validated experimentally by cell growth and spheroid formation assays. The current study provides evidence for the reprogramming of the co-regulation network between carbon metabolism and cancer pathway genes in HCC. In addition, this study also reveals a unique 22-carbon-metabolism-gene-expression-signature in HCC. Strategies targeting these genes may represent new therapeutic approaches for HCC treatment.

## INTRODUCTION

An unique characteristic of cancer is altered energy metabolism, a result of cancer cell genetic instability and/or effects of the tumor microenvironment [1]. Studies of past decades have revealed complicated and fine-tuned metabolic switch in cancer cells. Thus, metabolic reprogramming is considered as an emerging hallmark of cancer [2]. Aerobic glycolysis, also known as Warburg effect, is one of the predominant phenomena observed in malignant, rapidly growing tumor cells, which is characterized by a much higher glycolytic rate compared to their normal tissues of origin even if oxygen is abundant [3]. Like glycolysis, mitochondrial respiration is also required for tumor progression [4]. These carbon metabolic alterations can provide cancer cells not only energy but also substances used for synthesis of macromolecules, which are essential for cell proliferation and replication. Furthermore, the metabolic switch may confer a selective growth advantage that drives tumorigenesis. All of these aspects underscore the importance of metabolic reprogramming, a common phenomenon observed across multiple types of cancers including hepatocellular carcinoma (HCC) [2, 5].

In addition to metabolic switch in liver cancer, the liver itself is a hub of metabolism of human. Recently, considerable research efforts have been made to identify metabolic markers for the diagnosis and prognosis of HCC by using the strategy of high-throughput methods such as microarray, transcriptomics, and metabolomics [5]. Studies have been carried out to compare certain metabolites or gene expression levels between tissues or biofluids from patients with HCC or nonmalignant controls [5, 6]. Whereas high-throughput analyses have contributed to a better understanding of global regulatory sceneries in cancer cells, these methods also incurred additional complexity to results interpretation. Thus, it was not uncommon to see indecipherable, controversial and even conflicting results across these studies [6]. To better understand the regulatory network of metabolism in HCC, more systematic methods beyond simply comparing the levels of genes or the concentrations of molecules between malignant and non-malignant biomaterials are required.

Gene correlation network analysis, also known as gene coexpression network analysis, is a group of methods to systematically analyze large, high-dimensional gene expression data sets. Correlation networks are constructed on the basis of correlations between quantitative measurements of certain characteristics of elements included in a specific state [7]. Gene correlation network analysis is being increasingly utilized in bioinformatics applications, and several elegant studies have demonstrated its power in analyzing networks based on gene expression profiles in diverse areas including macrophage activation [8], key regulator identification in glioblastoma [9], and genetic programming of embryos [10].

In the current study, we constructed correlation networks of basic metabolism related genes using gene expression data from publicly accessible resources and defined gene clusters that have a close association with HCC. In parallel, we performed RNAseq analysis in human HCC and non-tumor liver tissues which revealed the spectrum of carbon metabolism pathway genes that are differently expressed between HCC and non-tumor liver tissues. The transcriptomics analysis enabled us to identify a unique 22-gene signature of carbon metabolism, whose expression levels are elevated in HCC tissues. Increased expression of these 22 genes in HCC was further verified in multiple microarray data sets. Analysis of The Cancer Genome Atlas (TCGA) database showed that the expression status of these 22 carbon metabolism genes is closely associated with the overall survival of HCC patients.

## RESULTS

### Network analysis indicates basic metabolic alteration in HCC tissues

**Figure 1:**
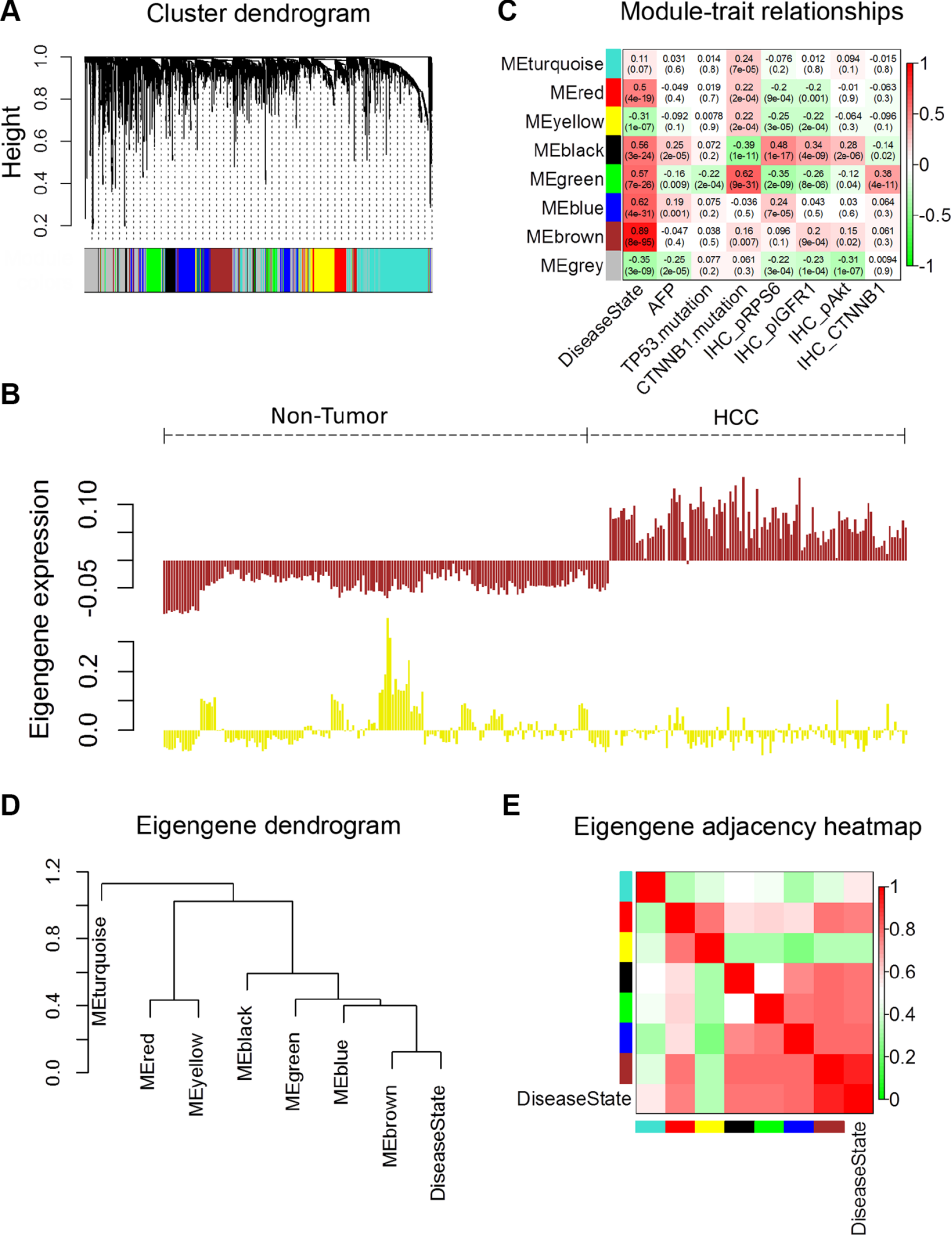
Weighted Correlation Network Analysis of metabolism related gene expression profiles of HCC and non-tumor tissues A total of 1509 genes (represented by 2804 probes in Affymetrix U133Plus2.0 array) were analysed by WGCNA. (**A**) Hierarchical clustering of metabolism related genes based on gene co-expression pattern across all samples in microarray data Set-1. Identified co-expression modules were represented by color classifiers (grey color is assigned to genes that are not part of any module). The y-axis height reflects the closeness of individual genes, which were represented by colored bars along x-axis. (**B**) Module eigengene patterns of genes grouped in brown and yellow cluster. The brown module has a strong positive correlation with HCC since its eigengene levels are consistently high across all HCC samples; while the eigengene levels of yellow module are low in majority of HCC samples, it has a moderate negative correlation with HCC. See also Figures S1A. (**C**) Module-trait associations. Each row corresponds to a module eigengene (ME), and each column to a trait. Each cell contains the corresponding correlation and *p*-value. The table is color-coded by correlation according to the color legend. The Alpha-fetoprotein (AFP) data shown here were the logarithmic transformations of the original values. (**D**, **E**) Visualization of the eigengene network representing the relationships among the modules and the clinical trait Disease State (HCC or non-tumor). Panel D shows a hierarchical clustering dendrogram of the eigengenes in which the dissimilarity of eigengenes *E_I_*, *E_J_* is given by 1 – cor(*E_I_*, *E_J_*). Panel E shows the heatmap of the eigengene adjacency *A_IJ_*, which defined *A_IJ_* = [1 + *cor*(*E_I_*, *E_J_*)]/2 (7). The color bars on left and below indicate the module of each row or column.

To investigate metabolic gene sets that are associated with the HCC status and their clinical traits, we applied WGCNA, which defines transcriptional modules based on Pearson correlation and determines relationship between these modules and different clinical traits [7]. The microarray data Set-1 was used for this analysis. First, expression information of 1509 genes (represented by 2804 probes in Affymetrix U133Plus2.0 array, as shown in Supplementary Table S1) involved in primary and secondary metabolic process (Supplementary Table S2) were selected and used as the input data for WGCNA. We identified 8 distinct coexpression modules containing 87 to 495 genes per module (Figure 1A and Supplementary Table S3). The expression data from different genes within each calculated module were used to determine the module eigengenes (i.e. the first principle component of the respective module), and the expression of eigengenes of each module was visualized, as shown in Figure 1B and Supplementary Figure S1A.

In order to identify modules that are significantly associated with the clinical traits, we correlated eigengenes with external traits and searched for the most significant associations. The resulting module-trait correlation was then visualized as a heatmap (Figure 1C). We observed that the disease state (HCC versus non-tumor) showed the most significant correlation with several modules. Based on the correlation coefficients, genes clustered in brown, blue, green, black and red modules are highly expressed in HCC tissues, while genes in yellow and grey modules are decreased in HCC tissues. Genes clustered in brown modules have the strongest positive correlation with patients' disease state, while genes in yellow module have negative correlation with the disease status. The gene significance vs. module membership plot of genes in brown module are shown in Supplementary Figure S1B; the data indicate that the genes highly significantly associated with the patients' disease state (HCC) are also the most important (central) elements of this module.

Next, we used the eigengenes as representative profiles to quantify module similarity and to determine their correlation with the disease status (HCC or non-tumor) by way of eigengene correlation. The dendrogram (Figure 1D) indicates that brown, blue, green and black modules are highly related with each other and also have strong correlations with disease state. Detailed eigengene adjacency of all modules and disease state are shown as a heatmap in Figure 1E. Taken together, this network analysis indicates that the expression levels and regulation of metabolism-related genes are altered in HCC compared to the non-tumor liver tissues.

### RNAseq based pathway mapping reveals a 22 carbon metabolism gene pattern in HCC

**Figure 2:**
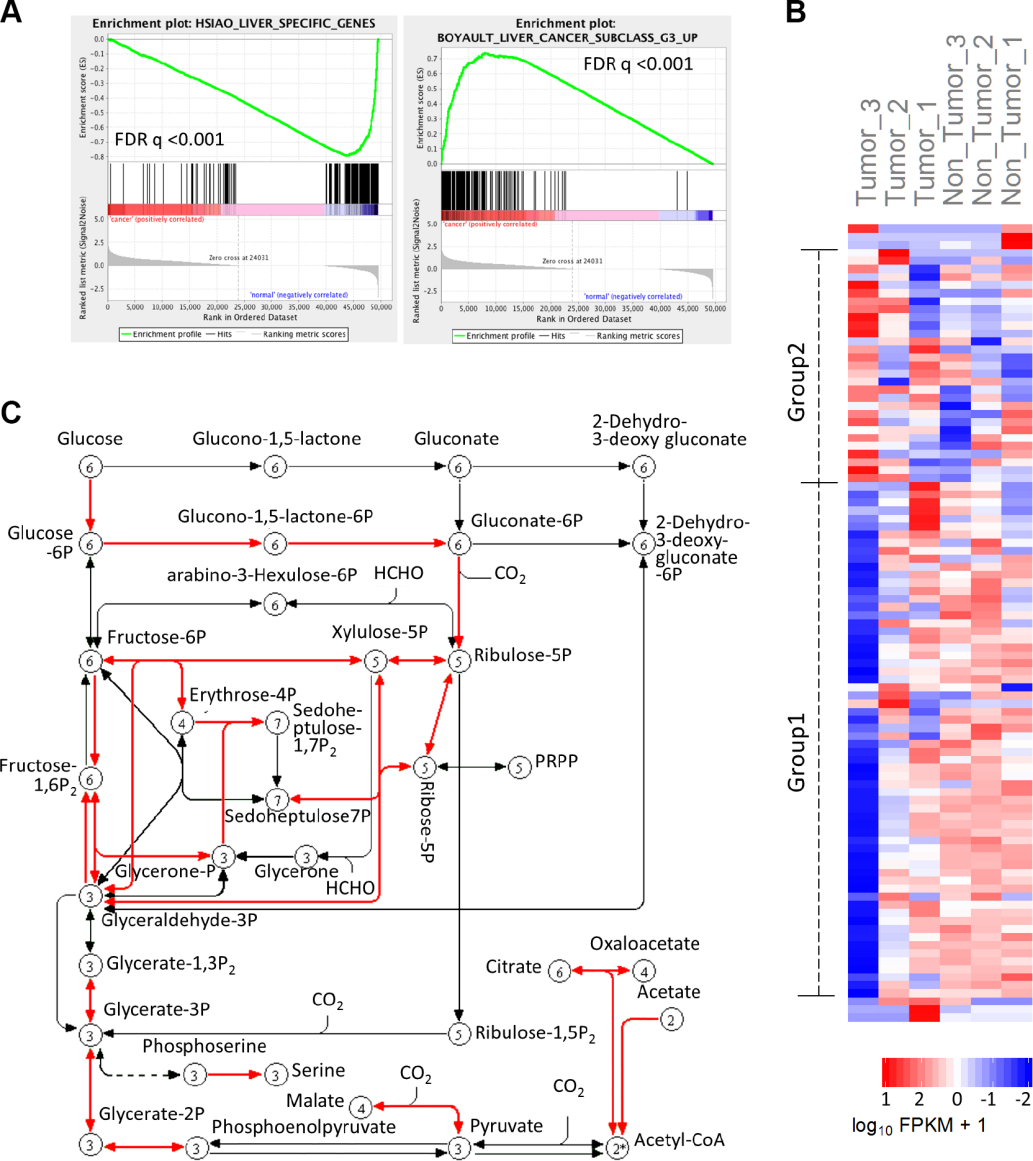
Transcriptome analysis shows carbon metabolism genes spectrum in HCC After mapping genes in different metabolism pathways to RNA sequencing data, carbon metabolism genes were found to have distinct expression pattern in HCC compared to non-tumor tissues. (**A**) GSEA results show that the levels of liver specific genes are decreased, while liver cancer related genes are increased in HCC tissues. (**B**) Heatmap shows the expression of carbon metabolism genes in 3 pairs of HCC and adjacent liver tissues. Tumor-3 pair has the most distinctive expression pattern of carbon metabolism genes. (**C**) Pathway map (adapted from KEGG) shows that most of the 22 up-regulated carbon metabolism genes catalyze reactions in glycolysis, with a small numbers of genes implicated in TCA cycle, acetyl-CoA synthesis and other reactions.

To identify altered metabolic pathways in HCC, we performed next generation sequencing analysis by Illumina HiSeq2000 using human hepatocellular carcinoma and matched non-tumor liver tissues. This approach allowed us to obtain transcriptome data from three paired HCC and non-tumor liver tissues. The transcriptome data were assembled by Cufflinks and then analyzed by additional programs. Specifically, the output from cufflinks were used as input files for GSEA analysis. The GSEA results show that many normal functional genes set in the liver (including liver specific genes, cytochrome-P450 related genes, and fatty acid metabolism genes) are down-regulated in HCC tissues, whereas genes associated with malignancy potential of hepatocellular cancers are up-regulated (Figure 2A and Supplementary Figure S2A); these data reflect the adequacy of the sequencing data and analysis. We separated genes belong to specific KEGG metabolism pathways, including carbohydrate, lipid and amino acid metabolism; the expression levels of genes in different pathways were analyzed and visualized as a heat map generated using the R cummeRbund package. We observed that the carbon metabolism (KEGG map number hsa01200) genes exhibit opposite expression patterns in HCC and non-tumor liver tissues. As shown in Figure 2B, the carbon metabolism genes can be roughly classed into two groups: Group 1 - lower level of expression in HCC (compared to non-tumor liver tissue); and Group 2 - higher level of expression in HCC (compared to non-tumor liver tissues). This phenomenon is more clearly depicted in Tumor-3 and non-Tumor-3 paired samples. It's worth mentioning that the Tumor-3 and non-Tumor 3 paired samples have the most dissimilar expression pattern of cancer related, key cellular signaling pathway genes (indicated as Pathways in Cancer by KEGG map hsa05200) among all the three sample pairs (Supplementary Figure S2B).

The carbon metabolism pathway includes 106 genes as per KEGG pathway hsa01200. Our results show that 22 out of the 106 genes were overexpressed (belonging to group2) in HCC compared to the non-tumor liver tissues (Supplementary Table S4) (the expression levels of these genes were also verified in the microarray datasets, as will be described in the following section). Furthermore, we found that 71% (27/38) of probes representing the 22 up-regulated genes were grouped in clusters with positive correlation with HCC (brown, blue, black and green modules in Figure 1C), while only 37% (60/162) of probes representing down-regulated or unchanged genes were grouped in the clusters with positive correlation with HCC. When mapping these 22 genes in carbon metabolism pathway, most of the genes are involved in glycolysis regulation, while others are related to tricarboxylic acid (TCA) cycle and acetyl-CoA production (Figure 2C).

In addition to the carbon metabolism pathway, expression of fatty acid degradation pathway (KEGG map hsa00071) genes were found to be down regulated unanimously in HCC; however, this pattern was not observed for genes of the fatty acid biosynthesis and elongation pathway (KEGG map hsa00061 and hsa00062, as shown in Supplementary Figure S2C and Supplementary S2D). These findings are consistent with the effect of hepatitis C virus on fatty acid metabolism in the liver, given that all of the three paired samples utilized for the RNAseq analysis were from patients with Hepatitis C virus (HCV) infection and that HCV is known to induce hepatic steatosis through inhibition of fatty acid degradation rather than fatty acid biosynthesis [11].

### Verification of the identified 22 carbon metabolism gene signature in microarray data

**Figure 3:**
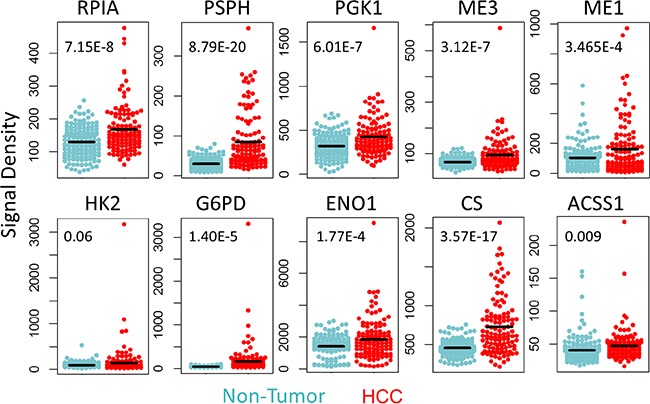
Up-regulated carbon metabolism genes verified in microarray data The expression levels of ten consistently up-regulated carbon metabolism genes in microarray data Set-1. The inserted numbers are the *P* values of student's *t*-test of expression levels for indicated genes.

We further analyzed the expression levels of the above-identified 22 carbon metabolism genes in microarray Set-1 and −2 (detected by 52 probes in Affymetrix U133Plus2.0 array). In Set-1, 37% (19/52) of probes were found to have a more than 1.2 fold increase in signal densities in HCC (compared to non-tumor liver tissues); 52% (27/52) of probes were found to have almost the same level as in non-tumor liver tissues (HCC:non-tumor ratio 0.83~1.2); only 11% (6/52) of probes have a decreased level (HCC:non-tumor ratio < 0.83). In Set-2, the percentages of probes with signal densities increased, no change, or decreased are 56% (29/52), 38% (20/52), and 6% (3/52), respectively. Ten consistently up-regulated genes across different data sets were shown as representatives in Figure 3 (Set-1 data) and Supplementary Figure S3 (Set-2 Data).

### Network analysis to determine gene associations by BioLayout Express3D

**Figure 4:**
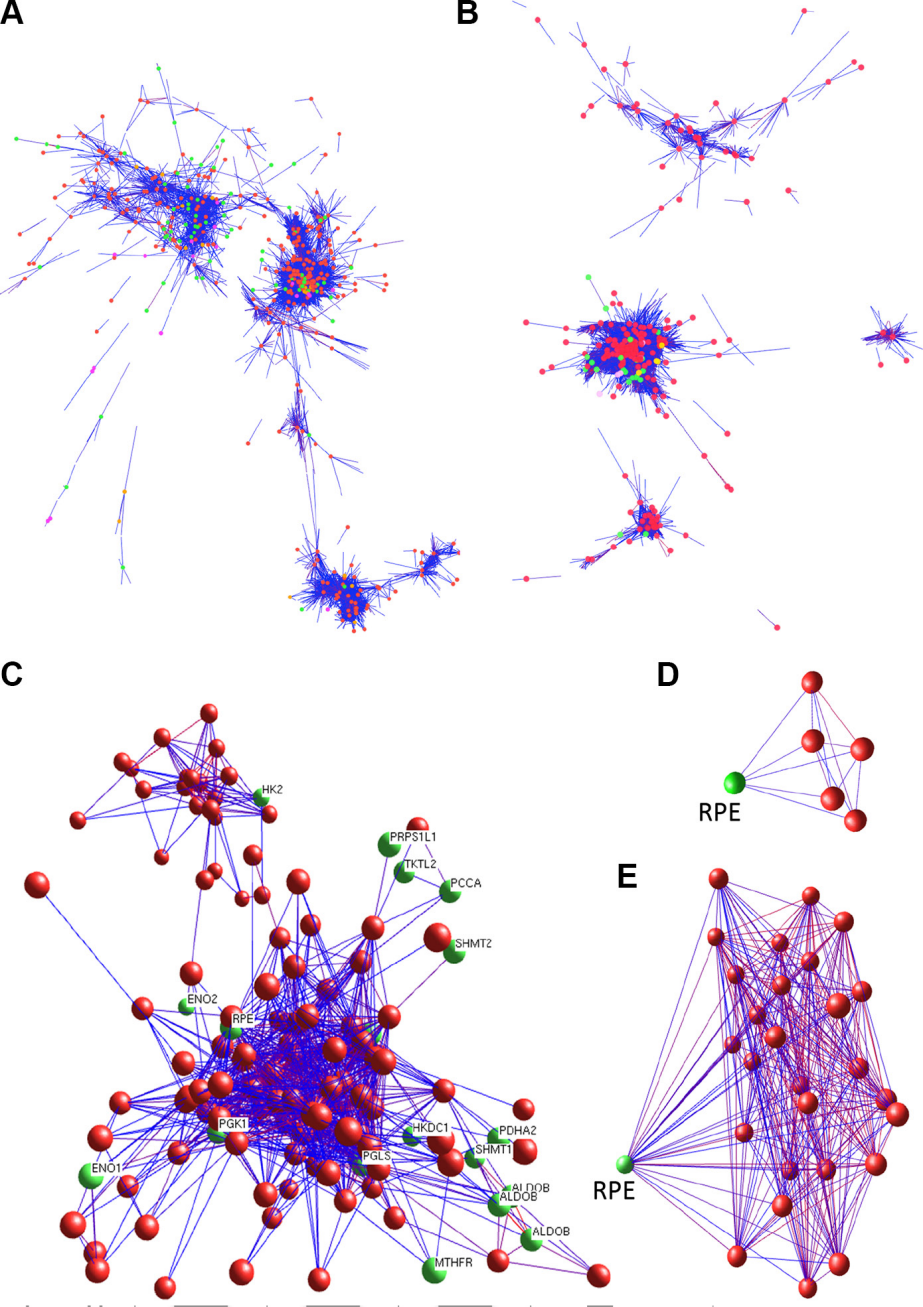
BioLayout Express3D analysis of genes involved in carbon metabolism and cancer cellular pathways Gene co-regulation analyses demonstrate that the co-regulation networks between the Carbon Metabolism and the Cancer Cellular Pathway genes are greatly altered in HCC. (**A**, **B**) whole co-regulation views of network show the interaction of genes of the carbon metabolism (green nodes) and the cancer cellular pathway (red nodes) in non-tumor (A) and HCC tissues (B). (**C**) detailed mapping shows the carbon metabolism genes with enhanced co-regulation of the cancer cellular pathway genes. (**D**, **E**) indicate gene RPE (green node) co-regulation network with cancer cellular pathway genes (red nodes) in non-tumor tissue (D) and HCC (E).

As described above, our RNA sequencing analysis revealed a distinctive gene expression pattern of both the Carbon Metabolism Pathway (Figure 2B) and the Pathway in Cancer (Supplementary Figure S2B) (especially in Tumor-3 and non-tumor 3 pair). These observations suggest the possible existence of a co-regulation network between these two pathway genes. This possibility was further evaluated by using BioLayout Express3D with microarray Set-1 as input data. Results of the BioLayout Express3D analysis show that, in non-tumor liver tissues, the majority of genes in these two pathways are well co-regulated and there exists an intertwined edges network (Figure 4A), suggesting a complex association among genes of these two pathways. However, in HCC tissues, the gene association networks among genes of these two pathways were reprogrammed (Figure 4B). These observations suggest that the co-regulation mechanism in normal liver tissues may be interrupted during the processes of carcinogenesis (via deregulation of whole genome expression or genome instability). In general, the associations between Carbon Metabolism and Pathway in Cancer genes become weaken in HCC, whereas the associations between a number of genes are enhanced, especially those in the 22 up-regulated carbon metabolism genes group (such as HK1, PGK1, ENO1, PGLS, RPE) (Figure 4C). Notably, RPE (ribulose-5-phosphate-3-epimerase) is one of the Carbon Metabolism genes that show enhanced association with the Pathway in Cancer genes. RPE catalyzes the interconversion between D-xylulose 5-phosphate and D-ribulose 5-phosphate; the later can be further converted to ribose 5-phosphate, a substrate and important determinant of the rate of de novo purine synthesis [12, 13]. Our data showed that RPE's co-regulation relationships with cancer pathway genes were shifted from moderate in non-tumor tissue (Figure 4D) to an enhanced situation in HCC (Figure 4E); it is conceivable that such an alteration may lead to more production of ribose 5-phosphate substrate for cancer cell proliferation.

### CS and/or ACSS1 genes knocked-down decrease HCC cells malignancy

**Figure 5:**
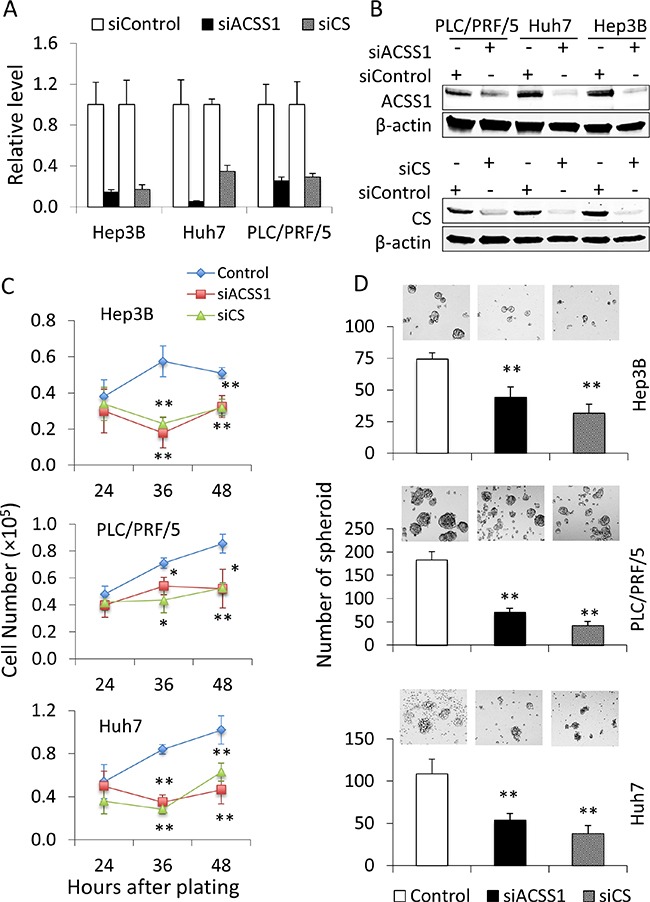
Knock-down of CS or ACSS1 is able to decrease the malignant characteristics of HCC cells HCC cell lines Hep3B, PLC/PRF/5, and Huh7 were transfected with CS- or ACSS1-specific siRNA; 24 hours after transfection, the cells were assessed for knockdown efficacies. (**A**, **B**) knockdown efficacies as determined by quantitative RT-PCR (A) and Western blot (B). (**C**) Cell proliferation was measured at 24, 48, and 72 hours in indicated cells after plating 5 × 10^4^ cells per well in 6-well plates. Knockdown of CS or ACSS1 significantly decreased cell proliferation. (**D**) cell spheroid formation assays were performed in CS or ACSS1 knockdown and control HCC cells. Single-cell suspensions were plated at a density of 5 × 10^3^ cells per well in 24-well Ultra-Low Attachment Plates. The cells with CS or ACSS1 knockdown showed more than 50% reduction in the number of hepatospheroid compared with individual control cells after 7 day-culture in serum-free medium. The data are expressed as means ± SD. *P < 0.05, **P < 0.01 vs. corresponding control cells.

To verify the results of the above-described *in silico* analyses, we selected representative genes from the 22-gene panel to determine their functional impact in HCC cells. Citrate synthase (CS) and Acetyl-CoA synthetases short-chain family member 1 (ACSS1) were selected, as these two enzymes have fundamental functions in alternative acetyl-CoA conversion and/or TCA cycles [14–17]. Importantly, the enzyme activity of both CS and ACSS1 have been reported to be increased in HCC and these two enzymes catalyze the reactions that provide alternative sources of energy and substrates for multiple rapid proliferating cancer cells including HCC cells [18–20]. We utilized siRNA to knock down CS and ACSS1 in HCC cell lines including Hep3B, PLC/PRF/5, and Huh7. As shown in Figure 5A and 5B, the mRNA and protein levels of CS and ACSS1 were significantly decreased in cells transfected with the specific siRNAs. The cells with CS or ACSS1 depletion were then analyzed for their proliferation and spheroid formation capacity. We observed that knockdown of CS or ACSS1 significantly decreased the proliferation and hepatospheroid formation efficiency of all three HCC cell lines when compared to their respective controls (Figure 5C and 5D). These data indicate that inhibitions of CS or ACSS1 gene expression are able to partially reduce the malignant characteristics of HCC cells.

### Carbon metabolism gene expression pattern influences HCC patient survival

**Figure 6:**
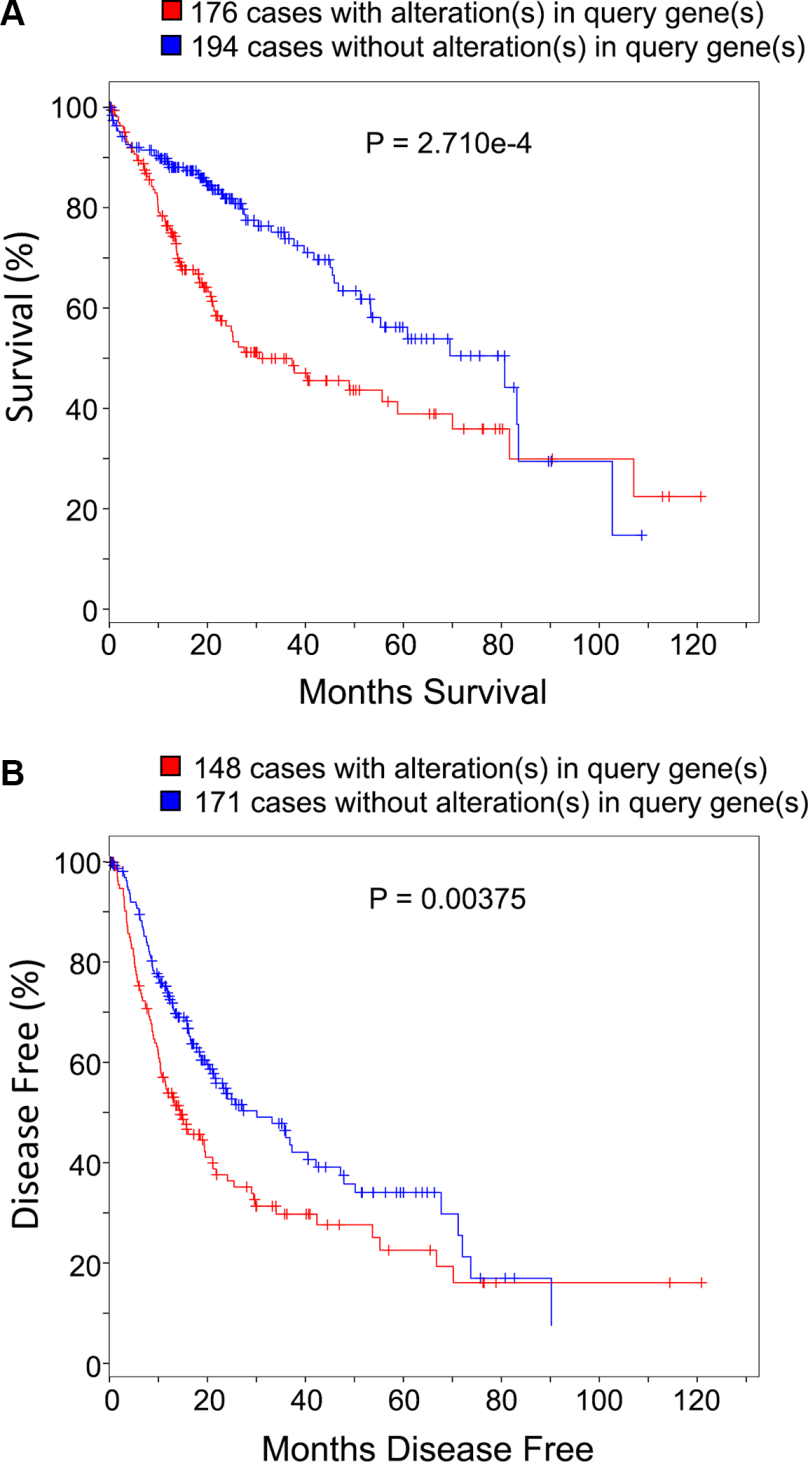
Carbon metabolism genes expression pattern influences HCC patient survival and prognosis The 22 up-regulated carbon metabolism genes were used as query genes for the online cBioPortal for Cancer Genomics tool of TCGA (http://www.cbioportal.org). There are totally 373 patients' clinical data as of 1^st^ December 2015. Panel (**A**) shows the Kaplan-Meier overall survival curve of HCC patients with or without alterations (up-regulated expression) in query genes. Panel (**B**) shows the Kaplan-Meier disease free survival curve of HCC patients with or without alterations (up-regulated expression) in query genes. *P* values on plots were derived from Log-Rank Test.

We next used publicly available data and tools from TCGA database to analyze whether the expression levels of the twenty-two carbon metabolism genes may influence HCC patients survival [21, 22]. Specifically, the 22 genes were used to query against the Liver Hepatocellular Carcinoma (TCGA, Provisional) data set, which includes both mRNA expression and survival information of 373 HCC patients as of the Eighteenth day of February 2016. Results of the analysis show that 47.2% (176/373) of HCC patients have gene expression altered in at least one of those 22 genes compared to the non-tumor liver tissues; 97.7% (375/384) of those alterations are up-regulated expression (Supplementary Figure S4). Of all the 176 patients in the TCGA provisional data set with altered expression of signature genes, the percentages of patients that have more than 2, 4, or 6 signature genes level increased are 47%, 20%, and 7.3% respectively. As combinations of increased genes vary among different patients, it is worth mentioning that the above-indicated systematic analysis between tumor and non-tumor tissues confers advantage over the traditional comparison of specific gene(s). Our analyses show that patients with one or more alterations of the 22 genes in their tumor tissues show shorter overall survival periods compared to patients without up-regulation of these genes (Figure 6A and Supplementary Table S5; median survival months: 30.58 *vs*. 80.68; Log-Rank Test *P* = 2.71 × 10^−4^). This trend is also observed in patients' disease free survival prognosis (Figure 6B and Supplementary Table S6; median disease free months: 14.22 *vs*. 29.96; Log-Rank Test *P* = 0.00375).

## DISCUSSION

Cells have a highly integrated network of mechanisms to coordinate metabolism with cellular functions; therefore, the metabolic state is constantly adjusted in response to extracellular signals or nutrient availability to meet the cellular activities [23]. In malignant cells, the co-regulation balance between metabolism and cell signaling is disturbed and rebalanced in the neoplastic environment. The regulation of the new equilibrium is complex in cancer cells, as many cancer-causing genetic alterations also regulate metabolism related genes [24]. In this study, we identified a group of 22 genes in the carbon metabolism pathway that are up regulated in HCC compared to the non-tumor liver tissues. Notably, 16 of 22 genes belong to key enzymes in the glycolytic pathway. Our findings are in support of the documented robust increase of glycolysis in hepatocellular carcinomas [25, 26]. Such a metabolic shift to glycolysis provides a selective growth advantage and also allows cancer cells to maintain mitochondrial bioenergetics and integrity during cell growth and proliferation [27]. Since mitochondrial is an essential source for both energy and biosynthetic substances [38], studies have documented the involvement of mitochondrial respiration-related genes in hepatocarcinogenesis [6, 29]. Here, we observed that HCC show increased expression of several genes who's products catalyze linkage reactions between glycolysis and tricarboxylic acid cycle, such as NADP^+^-dependent malic enzyme (ME), ACSS1, and CS.

Although originated from organ with sophisticated blood vascular network, studies have demonstrated that the arterial blood supply in HCC significantly decreases as the stage and histologic grade progress [30]. Thus, HCC cells might undergo metabolic stress in hypoxia and nutrient-poor conditions. Therefore, alterative carbon and energy resources are vital for cancer cells to survive and grow. Acetate, a 2-carbon fatty acid, is an alternative metabolic substrate and is readily converted to acetyl-CoA in multiple types of cancers including HCC [17, 18]. This conversion is catalyzed by acetyl-CoA synthetases short-chain family (ACSS) proteins. Studies have shown that ACSS activity contributes to acetate uptake regulation and cancer cell growth under low-oxygen condition. Indeed, the therapeutic feasibility of pharmacologically targeting ACSS is currently being explored [14]. In addition to ACSS, citrate synthase (CS), the first TCA cycle enzyme, is another potential target for cancer therapy [15, 16]. It is likely that enhanced CS activity contributes to the conversion of glucose to lipids in cancer by providing substrate for membrane lipid synthesis [15]. In our study, Both CS and ACSS1 gene expression were found increased in HCC tumor tissues. When ACSS1 or CS was knocked down by siRNA, HCC cells growth and spheroid formation were significantly reduced in low glucose circumstance. These results suggest that ACSS1 and CS may play an essential role in HCC, especially under relatively low blood supply condition in advanced disease (with hypoxia and low glucose).

Energy metabolism reprogramming is implicated in several key aspects of cancer cell biology, including cell survival/death, differentiation/proliferation and DNA repair [2]. A large numbers of studies have been carried out to evaluate the feasibilities of metabolite or metabolism related genes as potential diagnostic biomarkers or therapeutic targets of diverse types of cancers [5, 6, 31, 32]. Due to its long duration of carcinogenic process and progressive genomic instability, HCC shows great heterogeneity of genomic alterations which may contribute to the complexity of metabolic phenotypes [33]. Our current study provides global evidence for diverse metabolic alterations in HCC. From our RNAseq data, we observed that Tumor-3 has the most distinctive metabolic gene expression pattern among all sample. We reason that one possible explanation for the diverse metabolic profiles may relate to the differential expression of Hypoxia Induced Factor-1a (*HIF1α*) in the tumor tissues. We observed that *HIF1α* mRNA in Tumor-3 tissue was 6.7 times higher than its paired non-tumor liver tissues; in contrast, *HIF1α* mRNA only increased by ~1.5 folds in the tumor tissues of other two sample pairs. As HIF1α is one of the key molecules that regulate glycolysis and mitochondrial oxidative phosphorylation [34], it is possible that differential expression of HIF1α may contribute to the diversity of glucose metabolism in HCC.

Recent studies have reported alterations of metabolite or metabolism related genes in HCC [5, 6, 31, 32]. However, global alterations of metabolic gene networks have not been rigorously documented. To date, the majorities of published studies have only analyzed alterations of limited number of genes, and thus have not demonstrated the potential molecular diversity of metabolism alterations in HCC. Additionally, many studies did not show the ability to reproduce primary results consistently in diverse populations due to the heterogeneity of cancer metabolism [5]. Hence, the metabolic phenotype heterogeneity of HCC necessitates the use of systematic methods to provide a “macro perspective”. Such approaches are expected to systematically analyze metabolic gene expression profile and co-regulation network alterations and to facilitate the identification of metabolic signatures for clinical use [33]. In this context, the 22-carbon-metabolism-gene signature identified in our study may serve as a useful attempt to simplify the intricate and changeable metabolic dissimilarities between cancer and non-cancer cells. Therefore, the novel 22-carbon-metabolism-gene signature identified in our study may help further understand metabolic alterations in HCC and develop concepts regarding future prognostic and therapeutic approaches.

## MATERIALS AND METHODS

### Microarray datasets

Two microarray datasets for HCC and non-tumor liver samples gene expression generated by Affymetrix U133Plus2.0 array platform were identified and downloaded from public microarray data repositories ArrayExpress (European Bioinformatics Institute) and Gene Expression Omnibus (National Center for Biotechnology Information). Set-1, from ArrayExpress with accession E-MTAB-950 (https://www.ebi.ac.uk/arrayexpress/experim-ents/E-MTAB-950), includes 120 HCC and 160 non-tumor liver samples [35]; Set-2, combined GSE41804 [36], GSE17548 [37], GSE29721 [38], GSE33006 [39], GSE40873 [40], and GSE6222 [41] sets from GEO includes 60 HCC and 104 non-tumor liver samples. Most of the HCC patients in these two sets have the underlying etiology of Hepatitis C virus (HCV). All of the raw data were processed using affy and related R packages with Robust Multi-array Average approach for background normalization as per the package instruction.

### RNA isolation and sequencing

HCC and adjacent non-tumor tissue pairs were from three male patients with history of HCV infection, suffering from stage I or II hepatocellular carcinoma. Total RNAs were isolated from these frozen tissues with Qiagen RNeasy Mini kit. The quality of the isolated RNAs was monitored by Agilent Bioanalyzer. The RNA samples were polyA selected and processed to prepare sequencing libraries. The RNA-seq libraries were sequenced on Illumina HiSeq2000 instruments which generate an average of 50 million paired 75 bp reads per sample. The raw and analyzed RNA-seq data have been deposited in the Gene Expression Omnibus (GEO) database under accession number GSE81550.

### RNAseq data processing

Tophat-Cufflinks pipeline was used to map the qualified reads of each sample to human reference genome release GRCh37; the expression levels of each gene in HCC and non-tumor tissues groups were identified. All of the genes included in metabolic pathways were selected as per pathway gene lists from KEGG database (www.genome.jp/kegg/), mapped to differential expression results generated from Cuffdiff, and visualized as heatmaps by cummeRbund R packages.

### Gene Set Enrichment Analysis (GSEA)

Gene Set Enrichment Analysis was applied to the cufflinks results to determine gene expression pattern differences between HCC and non-tumor tissues. GSEA v2.1 software from Broad Institute was used for this purpose [42]; a ratio-of-classes metric for gene ranking and 1000 permutations of gene sets were used to study established collections of gene sets provided by the Molecular Signatures Database v5.0 (MSigDB, http://broad.mit.edu/gsea/msigdb). Gene sets with FDR < 0.25 were considered significant.

### Weighted Gene Coexpression Network Analysis (WGCNA)

Co-expression analysis was conducted using the freely accessible R package WGCNA (v 1.42) [7]. Instead of creating multiple networks and comparing their modules using all of the genes from microarray data sets, we used 1509 metabolic related genes (represented by 2804 probes in Affymetrix U133 Plus2.0 array) to construct the network. The module eigengene expression, adjacency matrix heatmap, Module-Trait relationships, and other related parameters/results were calculated and visualized as per the software instruction.

### Co-regulation analysis by BioLayout express3D

BioLayout Express3D (BioLayout) is a powerful tool for the visualization and analysis of network graphs [43]. We applied BioLayout to distinguish the different co-regulation status of genes involved in carbon metabolic and cancer pathway between HCC and non-tumor liver tissues. Correlation between all transcriptable genes was computed with a Pearson correlation cutoff of 0.85 for both HCC and non-tumor tissues. After constructing the networks of all probes on Affymetrix U133Plus2.0 array, only probes related to genes that belong to Pathway in Cancer (KEGG hsa05200) and Carbon Metabolism Pathway (KEGG hsa01200) are shown (to demonstrate the correlations and interactions of those two pathways in HCC and non-tumor tissues).

### Cell Culture and siRNA Transfection

Human HCC cell lines (Hep3B, Huh7, and PLC/PRF/5 cells) were obtained from ATCC (Manassas, VA) and cultured in Dulbecco's modified Eagle's medium (Invitrogen, Carlsbad, CA) with 10% fetal bovine serum (Sigma-Aldrich, St. Louis, MO) in a humidified atmosphere of 5% CO_2_. Specific siRNAs targeting citrate synthase (CS) or acetyl-CoA synthetases short-chain family member 1 (ACSS1) were purchased from Santa Cruz Biotechnology (Santa Cruz, CA), and transfected into cells with Lipofectamine^®^ 2000 (Invitrogen, Carlsbad, CA) according to the manufacturer's protocol.

### Quantitative Real-time Polymerase Chain Reaction (qRT-PCR)

Total RNAs were isolated from cells and reverse-transcribed. Quantitative PCR was performed by SYBR Green method. The primers used were: CS-forward, 5′-TGGCTAACACAGCTGCAGAA-3′; CS-reverse, 5′-CATAGCCTGGAACAACCCGT-3′; ACSS1-forward, 5′-GTGG GAGAGCCCATCAACTG-3′; ACSS1-reverse, 5′-AGATGCCACCTGTCTGCCAC-3′. β-ACTIN-forward, 5′-GTCGTCGACACGGCTCC-3′ and β-ACTIN-reverse, 5′-TCGTCG CCCACATAGGAATC-3′ were used as internal control.

### Western blotting

Logarithmically growing cells were lysed in RIPA buffer with protease inhibitors. After running SDS-PAGE gel, the proteins were transferred onto nitrocellulose membranes; the membranes were blocked and incubated with primary antibodies at 4°C overnight. Primary antibodies for CS or ACSS1 were purchased from Santa Cruz Biotechnology. IRDye 800CW or 680 LT labeled antibody were used as secondary antibodies. The membranes were scanned and quantified with the Odyssey^®^ Infrared Imaging System (LI-COR Biosciences, Lincoln, NE).

### Cell proliferation

Twenty-four hours after siRNA transfection, cells were plated in 6-well plates in triplicate with serum-free DMEM growth medium supplemented with 2 mM L-glutamine and 0.5 g/L glucose. Twenty-four, 48, or 72- hour after plating, cells were counted.

### Hepatic Spheroid Formation Assay

Single-cell suspensions were plated at a density of 5 × 10^3^ cells per well in 24-well Ultra-Low Attachment Plates (Corning, Tewksbury MA) and maintained in serum-free DMEM medium supplied with 2 mM L-glutamine and 1 g/L glucose at 37°C in a 5% CO_2_ humidified incubator for 7 days.

### Statistical analysis

Difference between groups was evaluated by SPSS 19.0 statistical software with one-way analysis of variance or student's *t*-test. Overall survival and disease-free survival were estimated and plotted using the Kaplan-Meier method; differences between groups were assessed by Log-Rank test. Other bioinformatics analyses including GSEA, WGCNA, and Co-regulation Analysis, were performed using software with default test statistics and cutoff values as specified in individual method sections. *P* value < 0.05 was considered as statistically significant.

## SUPPLEMENTARY MATERIALS FIGURES AND TABLES









## Abbreviations

ACSS1: Acetyl-CoA synthetases short-chain family member 1
CS: Citrate synthesis
ENO1: Enolase 1
GSEA: Gene Set Enrichment Analysis
HCC: Hepatocellular Carcinoma
HCV: Hepatitis C virus
HIF1α: Hypoxia Induced Factor-1a
HK1: Hexokinase 1
KEGG: Kyoto Encyclopedia of Genes and Genomes
PGK1: Phosphoglycerate Kinase 1
PGLS: 6-Phosphogluconolactonase
qRT-PCR: Quantitative RT-PCR
RNAseq: RNA sequencing
RPE: Ribulose-5-phosphate-3-epimerase
SDS-PAGE: Sodium dodecyl sulfate polyacrylamide gel electrophoresis
siRNA: Small interfering RNA
TGCA: The Cancer Genome Atlas
WGCNA: Weighted Gene Coexpression Network Analysis.
