# Supplementary material for: Gene network analysis reveals a novel 22-gene signature of carbon metabolism in hepatocellular carcinoma
Source: Oncotarget. 2016 Jun 23;7(31):49232–45. doi: 10.18632/oncotarget.10249 (PMC5226503; doi:10.18632/oncotarget.10249)
Supplement: Supplementary file 1 [file oncotarget-07-49232-s001.pdf]

Gene network analysis reveals a novel 22-gene signature of carbon metabolism in hepatocellular carcinoma

Supplementary Materials

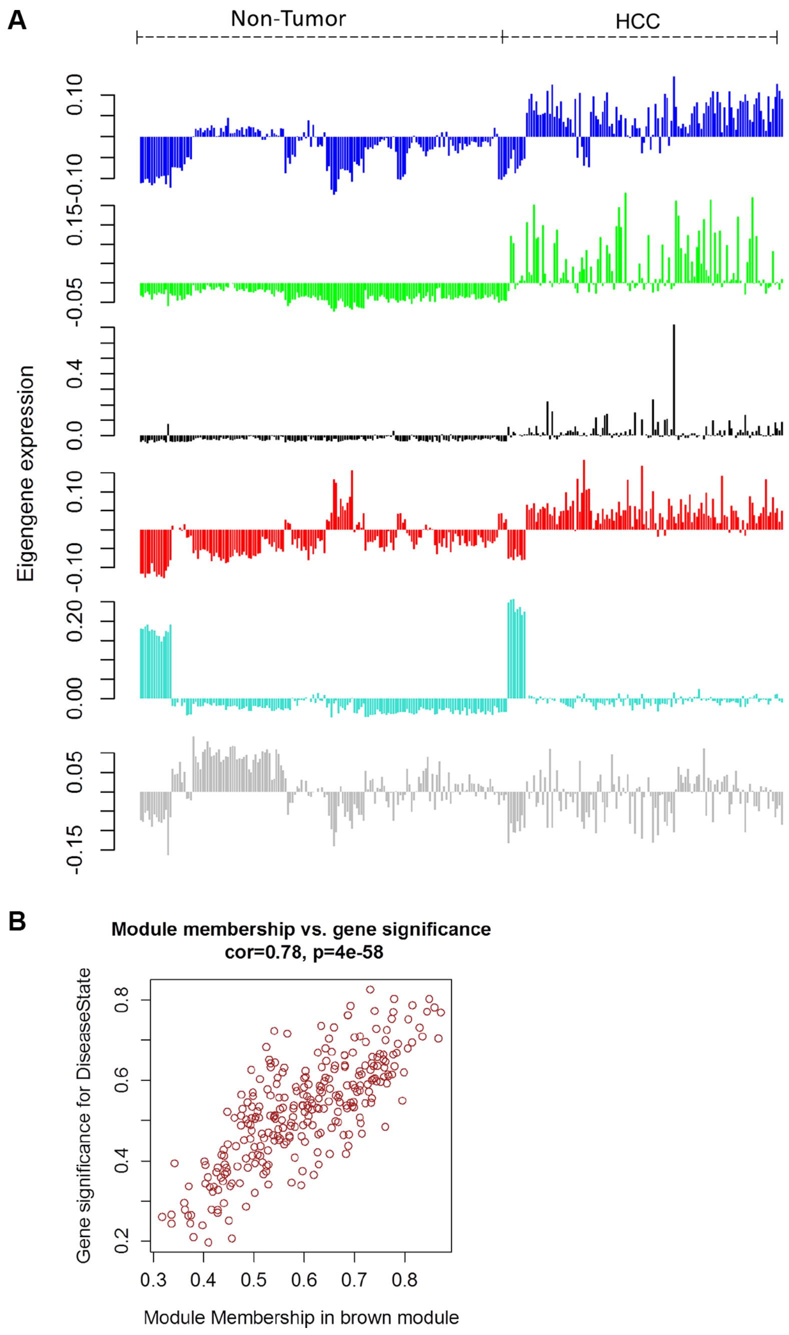

Supplementary Figure S1: (**A**) Module eigengene patterns of genes grouped in blue, green, black, red, turquoise and grey cluster. (**B**) Representative scatterplot of gene significance (GS) for weight vs. module membership (MM) in the brown module. There is a highly significant correlation between GS and MM in this module.

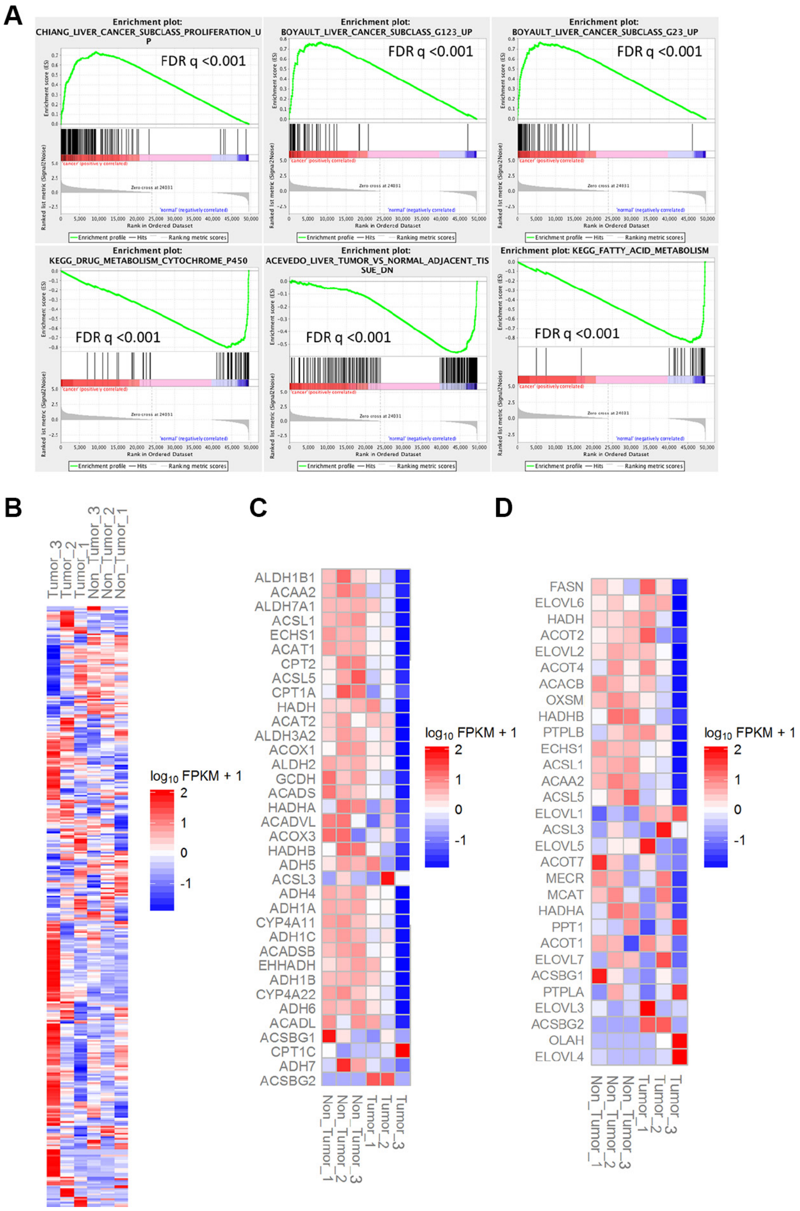

Supplementary Figure S2: GSEA results verify the high quality of our RNA sequencing data. (**A**) genes related to liver cancer are up-regulated, while genes involved in normal liver functions are down-regulated in HCC tissues. (**B**) heatmap shows the expression of cancer cellular pathway (KEGG hsa05200) genes in 3 pairs of HCC and adjacent liver tissues. Tumor-3 and non-tumor 3 pair has the most dissimilar expression pattern of these genes. (**C**, **D**) the heatmaps show expression level of genes of fatty acid degradation pathway (C) and fatty acid biosynthesis and elongation pathway (D). Almost all fatty acid degradation genes are found decreased in HCC, while such alteration was not observed for fatty acid biosynthesis and elongation genes.

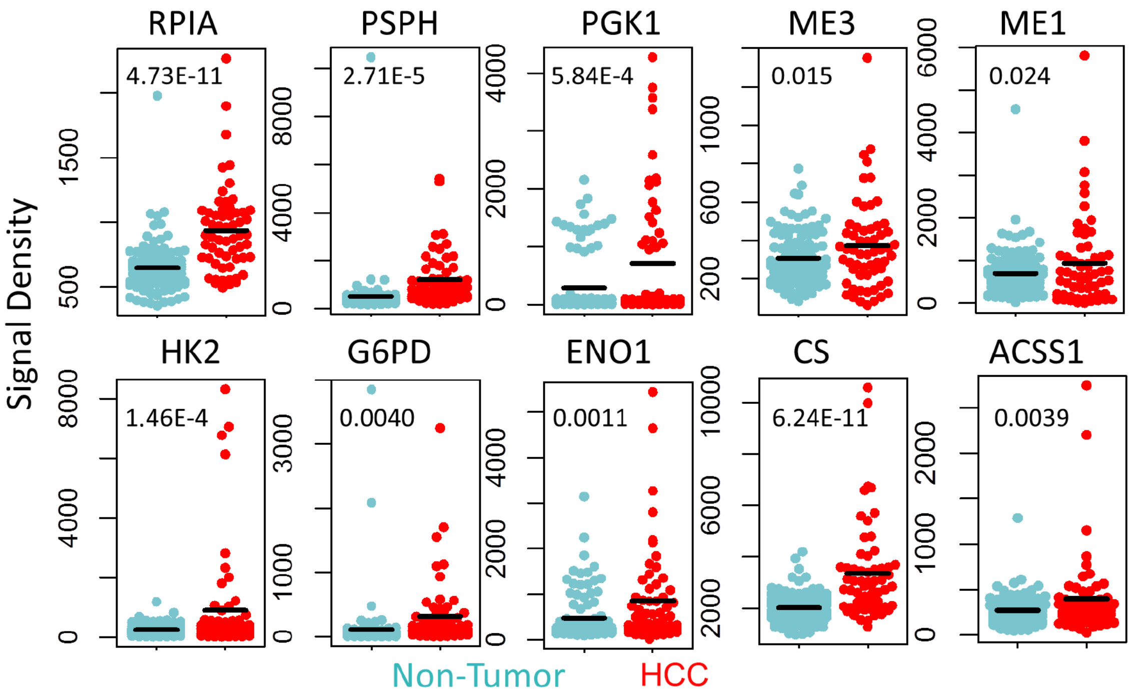

Supplementary Figure S3: Representative up-regulated genes included in the 22 carbon metabolism gene cluster were verified with microarray data Set-2. The inserted numbers are the *P* values of student's *t*-test of expression levels for indicated genes.

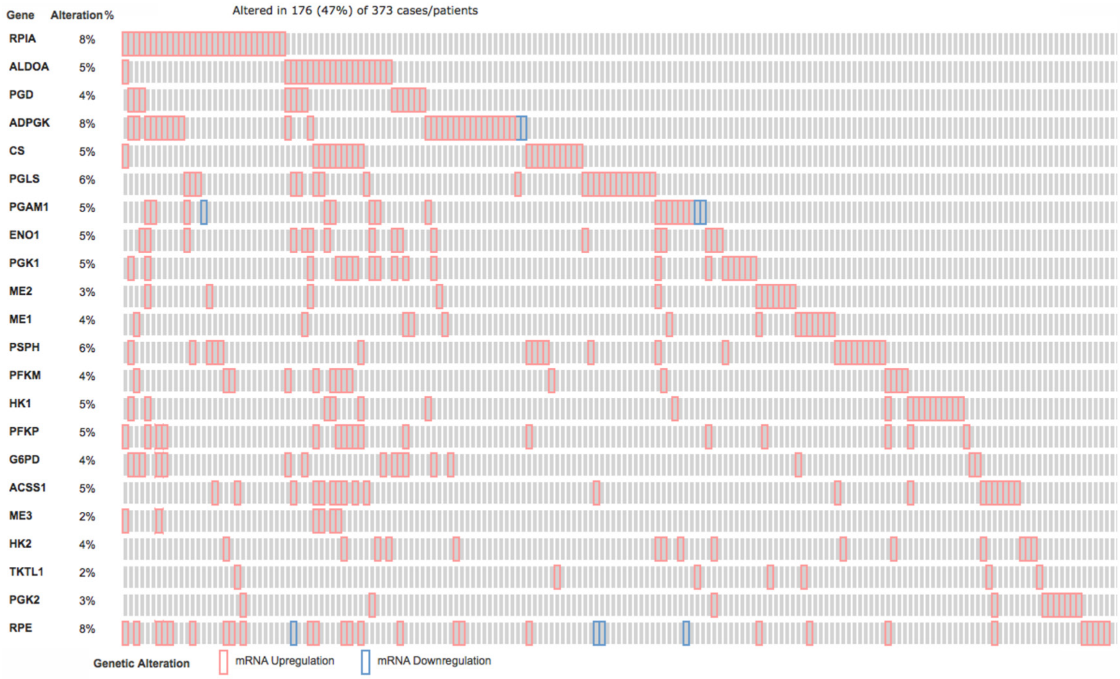

Supplementary Figure S4: The expression of 22 genes in HCC patients of the TCGA Provisional data set. 176 of 373 cases show altered expression of one or more genes included in the twenty-two gene signature; 97.7% (375/384) of those alterations are up-regulated expression. Each grey bar denotes one case of HCC patients.

Supplementary Table S1: Genes used in WGCNA network analysis. See Supplementary_Table_S1

Supplementary Table S2: Pathways

KEGG Pathway Number	KEGG Pathway Name	Catagroy
hsa00500	Starch and sucrose metabolism	Carbohydrate metabolism
hsa00620	Pyruvate metabolism	Carbohydrate metabolism
hsa00640	Propanoate metabolism	Carbohydrate metabolism
hsa00030	Pentose phosphate pathway	Carbohydrate metabolism
hsa00040	Pentose and glucuronate interconversions	Carbohydrate metabolism
hsa00630	Glyoxylate and dicarboxylate metabolism	Carbohydrate metabolism
hsa00010	Glycolysis / Gluconeogenesis	Carbohydrate metabolism
hsa00052	Galactose metabolism	Carbohydrate metabolism
hsa00051	Fructose and mannose metabolism	Carbohydrate metabolism
hsa00020	Citrate cycle (TCA cycle)	Carbohydrate metabolism
hsa00650	Butanoate metabolism	Carbohydrate metabolism
hsa00053	Ascorbate and aldarate metabolism	Carbohydrate metabolism
hsa00520	Amino sugar and nucleotide sugar metabolism	Carbohydrate metabolism
hsa00562	Inositol phosphate metabolism	Carbohydrate metabolism
hsa00190	Oxidative phosphorylation	Energy metabolism
hsa05230	Central carbon metabolism in cancer	Human Diseases
hsa00072	Synthesis and degradation of ketone bodies	Lipid metabolism
hsa00591	Linoleic acid metabolism	Lipid metabolism
hsa00564	Glycerophospholipid metabolism	Lipid metabolism
hsa00561	Glycerolipid metabolism	Lipid metabolism
hsa00062	Fatty acid elongation	Lipid metabolism
hsa00071	Fatty acid degradation	Lipid metabolism
hsa00061	Fatty acid biosynthesis	Lipid metabolism
hsa00565	Ether lipid metabolism	Lipid metabolism
hsa00590	Arachidonic acid metabolism	Lipid metabolism
hsa00592	alpha-Linolenic acid metabolism	Lipid metabolism
hsa00100	Steroid biosynthesis	Lipid metabolism
hsa00120	Primary bile acid biosynthesis	Lipid metabolism
hsa00140	Steroid hormone biosynthesis	Lipid metabolism
hsa00600	Sphingolipid metabolism	Lipid metabolism
hsa01040	Biosynthesis of unsaturated fatty acids	Lipid metabolism
hsa01200	Carbon metabolism	Metabolism

Supplementary Table S3: Modules of 1509 metabolic genes. See Supplementary_Table_S3

Supplementary Table S4: Genes included in KEGG pathway hsa01200. See Supplementary_Table_S4

Supplementary Table S5: Overall Survival of TCGA patients with or without alterations in 22 metabolism-related genes

	#Total cases	#Cases deceased	Median months survival
Cases with Alteration(s) in Query Gene(s)	176	76	30.58
Cases without Alteration(s) in Query Gene(s)	194	54	80.68

Supplementary Table S6: Disease Free Survival of TCGA patients with or without alterations in 22 metabolism-related genes

	#Total cases	#Cases relapsed	Median months disease free
Cases with Alteration(s) in Query Gene(s)	148	88	14.22
Cases without Alteration(s) in Query Gene(s)	171	86	29.96
